# Takotsubo cardiomyopathy in elderly female trauma patients: a case series

**DOI:** 10.1186/s13256-021-03056-1

**Published:** 2021-09-03

**Authors:** Vishal Patel, Shuli Levy, Iqbal Malik, Michael B. Fertleman, Louis J. Koizia

**Affiliations:** 1 Imperial College Healthcare NHS Trust, London, UK; 2 Cutrale Perioperative and Ageing Group, Department of Bioengineering, Imperial College London, London, UK

**Keywords:** Takotsubo cardiomyopathy, Trauma, Geriatrics, Frailty

## Abstract

**Background:**

Takotsubo cardiomyopathy is a syndrome characterized by acute left ventricular wall motion abnormalities leading to left ventricular systolic dysfunction. It remains an important differential diagnosis for acute coronary syndrome.

**Case presentations:**

Here we describe three cases of Takotsubo cardiomyopathy occurring in three Caucasian female trauma patients (aged 79, 81, and 82 years old) and the impact on their clinical course.

**Conclusions:**

For patients requiring surgical management, delays in the diagnosis of Takotsubo cardiomyopathy may lead to postponement of urgent operative management. This delay in surgery likely impacts on length of hospital stay, leading to an increasing morbidity and mortality.

## Background

Originally reported in Japan in 1990, Takotsubo cardiomyopathy is a syndrome characterized by acute left ventricular wall motion abnormalities with a hallmark transient apical ballooning leading to left ventricular systolic dysfunction [1, 2]. Commonly mistaken for an acute coronary syndrome (ACS), it can present with transient cardiac ischemia-like symptoms such as chest pain and shortness of breath with electrocardiogram (ECG) changes of ST segment elevation in the absence of significant coronary artery disease. Biochemical markers of cardiac injury (troponin I and T, creatinine kinase, and myoglobin) are usually elevated [2]. The clinical presentation can range from mild symptoms to severe pulmonary edema and cardiogenic shock [3]. With prompt recognition and management, there is usually a good prognosis and low mortality regardless of the severity of the clinical presentation [4].

The incidence of Takotsubo cardiomyopathy has been reported in around 2% of patients presenting with symptoms suggestive of ACS [5]. It appears to more common in people between the ages of 65 and 70 and in postmenopausal women [6]. Although the pathogenesis is not fully understood, there is evidence to suggest that sympathetic stimulation is integral [1, 7]. Increased plasma catecholamine levels in the setting of Takotsubo cardiomyopathy are usually precipitated by an event related to acute physical illness or emotional stress [7].

For patients presenting following trauma, in urgent need of operative management, the clinical conundrum relates to the concern that these patients are contemporaneously experiencing an ACS. The symptoms, signs, and investigations usually result in delays to operative management and heightened anxiety of the anesthetic risk. Differentiating Takotsubo cardiomyopathy from an ACS may prevent operative delays and avoid inappropriate management.

In this paper we present three cases of Takotsubo cardiomyopathy observed in elderly females admitted to a major trauma center following falls.

## Case presentations

### Case 1

A 79-year-old Caucasian woman with a past medical history of chronic obstructive pulmonary disease and ischemic heart disease (previous myocardial infarction in 2012 medically managed) was brought by ambulance to a major trauma center following presentation to her local emergency department with an accidental fall down 13 stairs at home. She reported feeling well prior to the fall and denied any central chest pain or shortness of breath on admission.

On examination, she was hemodynamically stable, with peripheral oxygen saturation of 88% and respiratory rate of 18 breaths per minute with a 24% oxygen requirement. Her arterial blood gas showed type 2 respiratory failure. She was found to have multiple left-sided rib fractures on an initial trauma computed tomography (CT) scan (2nd–8th with flail segments 4th–6th) and bilateral pretibial lacerations on physical examination.

**Fig. 1:**
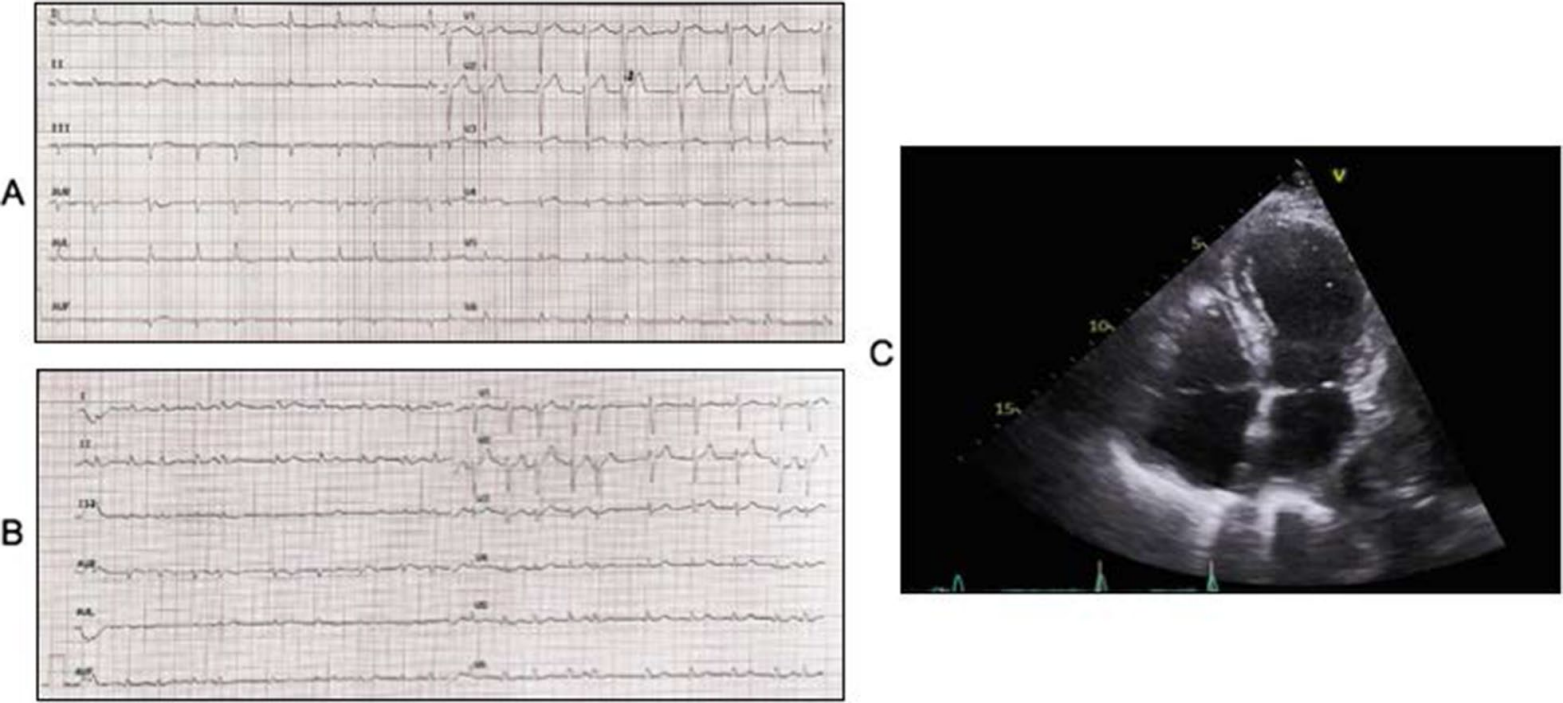
Admission (**a**) and discharge (**b**) electrocardiogram showing resolution of ST elevation in leads V2–V3 and echocardiography (**c**)

On admission, her troponin I was elevated at 2485 ng/L (normal < 16 ng/L), and ECG showed rate-controlled atrial fibrillation with ST elevation in leads V2–V3 (Fig. 1). Repeat troponin I was 1788 ng/L, and she was managed as for ACS, with dual antiplatelet therapy. An echocardiogram showed normal left ventricular size with severely impaired systolic function (estimated left ventricular ejection fraction [LVEF] 32%) with akinesis in the mid to apical segments and hypokinetic basal segments. B-type natriuretic peptide (BNP) was elevated at 2410 ng/L (< 266 ng/L).

Her rib fractures were managed with a thoracic epidural for 7 days and tibial lacerations sutured. Due to an increasing oxygen requirement, a CT pulmonary angiogram ruled out a pulmonary embolism, but showed a left pleural effusion which required chest drain insertion.

An inpatient CT coronary angiogram showed dense calcification of all three main coronary arteries which was very similar to previous angiography. A repeat echocardiogram prior to discharge showed improved left ventricular function, making the diagnosis of Takotsubo cardiomyopathy secondary to trauma. She was discharged following a 14-day inpatient stay on single antiplatelet therapy and anticoagulation for atrial fibrillation.

### Case 2

An 81-year-old Caucasian woman presented to the emergency department following a multifactorial fall onto her left side while mobilizing with a stick at home. Her past medical history included osteoporosis with a previous fragility fracture, asthma, myasthenia gravis, and previous thyroidectomy and thymectomy.

**Figure 2:**
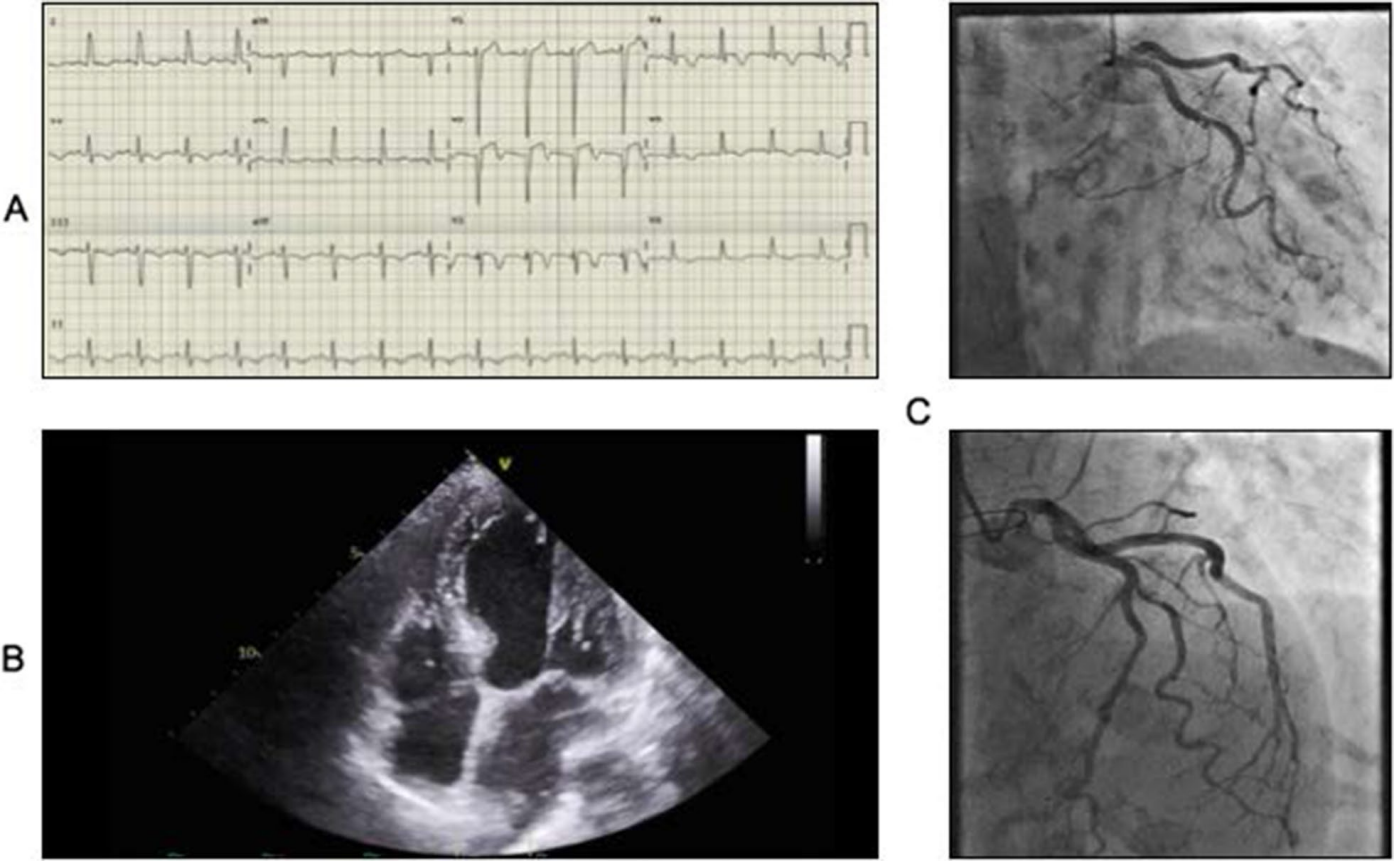
Admission electrocardiogram showing ST segment elevation in leads V1–2 (**a**), echocardiography showing septal bulge (**b**), and coronary angiography showing unobstructed coronary arteries (**c**)

On examination, she was tachycardic at 103 beats per minute, with a blood pressure of 131/77. She required 24% oxygen to maintain peripheral saturation of 93%. Bloods revealed a raised troponin I of 1080 ng/L and 2300 ng/L on repeat 4 hours later. BNP level was 924 ng/L. ECG showed a sinus tachycardia with septal Q-waves, ST elevation in V1–V2, and inferior T-wave inversion (Fig. 2). Hip X-ray revealed a left traverse fracture though the midshaft of the femur with proximal overlap requiring operative management. She had no chest pain. A bedside handheld echocardiography showed severe left ventricular impairment (LVEF < 25%) with anterior, anterolateral, and anteroseptal akinesia. She was managed as a high-risk ACS and given dual antiplatelet therapy. A departmental echocardiogram revealed normal left ventricular size with septal bulge and severely impaired systolic function (LVEF 30–35%).

The anesthetic team were concerned due to possible ACS and delayed operative management. She eventually went on to have definitive fixation (left closed reduction and femoral nailing) on day 6 of her admission with no perioperative complications. Her clopidogrel was held 24 hours prior to surgery (at the request of the surgeons), and restarted 3 days postoperatively.

Following the operation, she underwent an inpatient coronary angiogram at day 15. It showed mild atheroma in left anterior descending, left circumflex, and right coronary arteries with no occlusion. The patient was followed up in the cardiology clinic 6 weeks following her admission. She denied any chest pain and was at her baseline preadmission functional status. A cardiac magnetic resonance imaging (MRI) showed improvement in left ventricular function with an ejection fraction of 64%. Her BNP and troponin I levels had normalized by this time.

### Case 3

An 82-year-old Caucasian woman was brought in by ambulance as a primary percutaneous coronary intervention (PCI) call following findings of anterolateral ST elevation following an unwitnessed fall while at home with a long lie. Her past medical history included left-sided breast cancer (wide local excision May 2019), asthma, hypertension, osteoarthritis, and osteopenia.

On physical examination, she was tachycardic at 115 beats per minute with a blood pressure of 129/56 mmHg. There was no respiratory distress or oxygen requirement. She was noted to have a shortened and externally rotated left lower limb. Her ECG showed sinus tachycardia with ST elevation in leads V3–5 with Q waves. Bloods showed raised troponin of 12,428 ng/L, BNP of 1368 ng/L, impaired renal function, and raised creatinine kinase 2023 U/L. X-ray of the left hip showed a left subcapital neck of femur fracture. A bedside handheld echocardiogram showed apical akinesia and hyperdynamic basal-to-mid systolic function with mild systolic dysfunction. Regional wall motion abnormalities did not follow a typical coronary distribution. Her working diagnosis by the reviewing cardiologist was Takotsubo cardiomyopathy, and she continued on single antiplatelet therapy. She did not undergo formal coronary angiography.

**Fig. 3:**
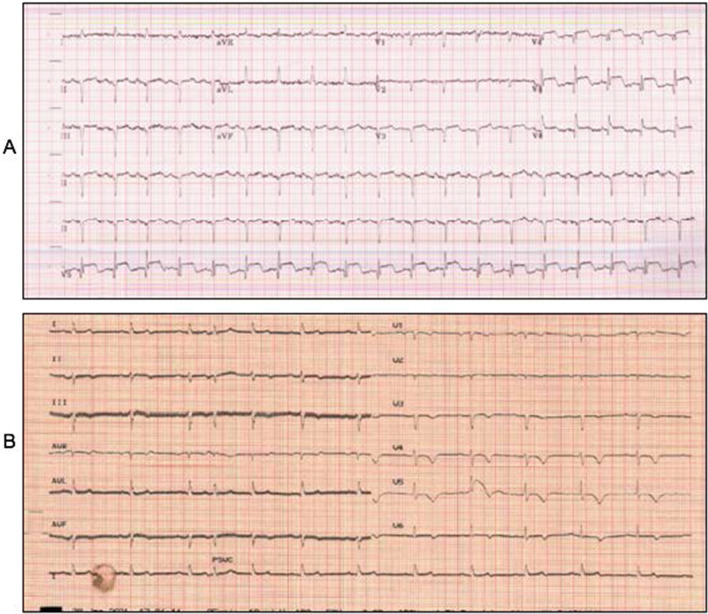
Electrocardiograms of patient 3 performed on admission (**a**) and on discharge (**b**) showing resolution of ST elevation in leads V3–V5

She had an uneventful left bipolar cemented hip hemiarthroplasty 48 hours following admission. Postoperatively, her echocardiogram showed improved biventricular function. Her ECG showed T wave inversion and resolution of ST segment elevation (Fig. 3).

## Discussion

In our case series, the patients fulfilled the criteria for the diagnosis of Takotsubo cardiomyopathy based on a precipitating physical stressor in the setting of trauma as a trigger, elevated troponin levels, ST segment changes on ECG, and reversible left ventricular dysfunction on echocardiogram, with recovery prior to discharge. These characteristics are part of the InterTAK diagnostic score which assesses the diagnostic likelihood of Takotsubo cardiomyopathy [8]. The demographics of our cases are in line with previous reports where 90% of cases occur in females with the majority over the age of 60 [9].

Emotional stressors account for approximately 15–40% of cases of Takotsubo cardiomyopathy, whereas physiological triggers such as acute medical illness or surgery have been reported in up to 70% of cases [10]. In elderly patients presenting following trauma, Takotsubo cardiomyopathy may be multifactorial. In addition to the trauma causing emotional distress, the peri- and postoperative phases and underlying medical illness may act as physiological stressors, particularly in elderly patients with multiple comorbidities [11].

The etiology of Takotsubo cardiomyopathy is not fully understood. Several hypotheses derived from observational studies have suggested the importance of the vascular and myocardial response to stress. Initially, multivessel coronary vasospasm was thought to explain the acute ventricular dysfunction seen in Takotsubo cardiomyopathy. However, in the majority of cases, there was no reported spontaneous coronary artery spasm or inducible vasospasm in follow-up studies [12–14]. Intravascularly, there appears to be no association between Takotsubo cardiomyopathy and acute atherosclerotic plaque rupture seen in ACS [15].

In patients with Takotsubo cardiomyopathy, catecholamine levels in the blood were found to be approximately ten times higher than normal, suggesting the importance of catecholamines in mediating cardiac injury [16]. This is also supported by conditions associated with raised catecholamines such as pheochromocytoma and thyrotoxicosis being associated with Takotsubo cardiomyopathy [17, 18]. An increased sensitivity of beta-adrenergic receptors to levels of catecholamines in the myocardium during periods of stress may be responsible for the pathophysiological findings in Takotsubo cardiomyopathy, particularly in the apical myocardium where higher densities of beta-adrenergic receptors are found [19]. Estrogen usually leads to a suppression of beta-adrenergic receptor expression in the ventricular myocardium [20]. In postmenopausal women, the loss of estrogen leads to an increased response to beta-adrenergic receptor agonists, which may account for the gender differences in the incidence of Takotsubo cardiomyopathy.

Previous cases have been reported of patients with Takotsubo cardiomyopathy in the setting of trauma [21, 22] and in elderly patients with hip fractures requiring operative management [14, 23]. Optimal timing of urgent surgery in the setting of Takotsubo cardiomyopathy is not known, with ranges from 48 hours to 6 days post admission, as seen in our cases. In both our cases associated with hip fractures, patients made a full recovery despite differing time to surgical fixation. Delays in surgery may lead to increasing analgesia requirements, increased hospital-acquired infections, and delayed mobilization and rehabilitation, which may contribute to a prolonged length of hospital stay and potential increased mortality [24]. The Best Practice Tariff for hip fractures was introduced to ensure that operative management of these fractures occurred within 36 hours of admission [25].

Patients with Takotsubo cardiomyopathy who are admitted with raised troponin levels and ECG changes of ST elevation, as in our cases, are often initially managed as ACS and given dual antiplatelet therapy. This is of particular importance in patients presenting following trauma and in those requiring surgery [11, 23]. Takotsubo cardiomyopathy has also been reported in patients following brain trauma [26–28], with the majority of ECG changes (ST segment deviation, T wave abnormalities, QT prolongation) being reported in patients with severe subarachnoid hemorrhage [29]. In these patients, management with dual antiplatelet therapy, including clopidogrel, may contribute to a further delay of urgent surgery and increased risk in perioperative bleeding, increased need for blood transfusions, and infection [30].

To combat this, prompt assessment by a cardiologist and early echocardiography may help in reaching an early diagnosis of Takotsubo cardiomyopathy, avoiding delay to urgent surgical management. While coronary angiography is the optimal imaging modality for excluding high-grade coronary stenosis, in some patients, this may not be suitable. In patients who are frail with life-threatening comorbidities, invasive angiography may be associated with considerable risk [6]. Noninvasive CT coronary angiography (CTCA) may be considered an alternative imaging modality in these patients. A study in 11 patients with suspected Takotsubo cardiomyopathy showed use of a contemporary CTCA ruled out significant coronary artery disease in 80% of patients [31]. Further studies are required to determine whether it can be used early in the management of Takotsubo cardiomyopathy to prevent delays in patients requiring surgery.

The management of Takotsubo cardiomyopathy is based upon identifying patients with adverse features secondary to complications. For patients with hypotension and cardiogenic shock, prompt assessment of whether left ventricular outflow obstruction exists will determine the suitability of inotropic support [6]. Beta-blockers have not been shown to reduce mortality in patients with heart failure secondary to Takotsubo cardiomyopathy and should be avoided in patients with bradycardia and QTc prolongation due to the risk of pause-dependent torsades de pointes [32, 33]. For patients presenting with acute heart failure, diuretics and prognostic medical therapy (angiotensin-converting enzyme inhibitors, aldosterone antagonists) are the mainstay of management. The majority of patients will present without complications, in which case the management is supportive and prognostic medical therapy can be considered [32]. Their impact on prognosis and recurrence of Takotsubo cardiomyopathy is not fully known. Antiplatelet therapy has been shown to reduce major adverse cardiovascular events during hospitalization [34]; however, the impact of its long-term use is unknown, particularly in elderly patients with Takotsubo cardiomyopathy [32].

## Conclusion

Since its initial reports in 1990, Takotsubo cardiomyopathy is being increasingly reported, particularly in elderly females following trauma. The severity of this syndrome ranges from mild symptoms, as presented in our cases, to life-threatening cardiogenic shock and pulmonary edema. It remains an important differential diagnosis for ACS. For patients requiring surgical management, delays in the diagnosis of Takotsubo cardiomyopathy may lead to postponement of urgent operative management. This may impact on length of hospital stay, time to recovery, as well as increased morbidity and mortality, not only due to the delays but to the use of antiplatelet medication. To aid the prompt diagnosis of Takotsubo cardiomyopathy in patients requiring surgery, we advocate a multidisciplinary approach with combined decision making from physicians, cardiologists, anesthetists, and surgical teams.

## Data Availability

N/A.
